# Cooperation in an Uncertain World: For the Maasai of East Africa, Need-Based Transfers Outperform Account-Keeping in Volatile Environments

**DOI:** 10.1007/s10745-016-9823-z

**Published:** 2016-05-03

**Authors:** Athena Aktipis, Rolando de Aguiar, Anna Flaherty, Padmini Iyer, Dennis Sonkoi, Lee Cronk

**Affiliations:** Arizona State University, Tempe, AZ 85281 USA; Rutgers University, New Brunswick, NJ USA

**Keywords:** Need-based transfers, Account-keeping transfers, Risk pooling, Herd survival outcomes, Maasai, East Africa

## Abstract

Using an agent-based model to study risk-pooling in herder dyads using rules derived from Maasai *osotua* (“umbilical cord”) relationships, Aktipis *et al*. (2011) found that osotua transfers led to more risk-pooling and better herd survival than both no transfers and transfers that occurred at frequencies tied to those seen in the osotua simulations. Here we expand this approach by comparing osotua-style transfers to another type of livestock transfer among Maasai known as *esile* (“debt”). In osotua, one asks if in need, and one gives in response to such requests if doing so will not threaten one’s own survival. In esile relationships, accounts are kept and debts must be repaid. We refer to these as “need-based” and “account-keeping” systems, respectively. Need-based transfers lead to more risk pooling and higher survival than account keeping. Need-based transfers also lead to greater wealth equality and are game theoretically dominant to account-keeping rules.

## Introduction

People everywhere have to deal with both predictable and unpredictable risks to their livelihoods. From modern day humans grappling with uncertainties about health and employment, to Maasai pastoralist managing large herds in the face of potential drought, disease and theft, risk management is an important adaptive problem for humans around the world. Humans use many strategies to manage risk, including risk retention (accepting risk and absorbing losses), risk avoidance (reducing dependence on high variability outcomes), risk reduction (lowering the probability of or size of losses) and risk transfer (moving risk from one party to another) (Dorfman 2007). Risk transfer is of particular interest to social scientists because it is the only one of these methods that requires cooperation. One common form of risk transfer is risk-pooling (also referred to as risk-sharing). Here we use two different types of livestock transfer found among Maasai pastoralists in East Africa to examine whether some rules regarding such transfers lead to more risk-pooling and more effective risk management than others.

We build upon Aktipis *et al*. (2011), which used an agent-based model to examine the impact of stock friendships on risk-pooling and herd survival among East African pastoralists. That model was based specifically on the rules underlying Maasai stock friendships, which they refer to by their word for umbilical cord: *osotua*. The rules that govern osotua relationships are straightforward: Ask only if you are in need and only for what is needed, and give if you are able to do so without threatening your own survival. The model showed that pairs of herders that follow the rules of osotua had herds that survive longer in the face of occasional shocks than pairs that either engage in no livestock exchange or that exchange livestock probabilistically at rates derived from the osotua simulations. In this article, we expand our understanding of dyadic livestock exchange among Maasai by comparing osotua transfers with transfers Maasai refer to as *esile*, which translates simply as “debt” (Mol 1996).

Maasai and other Maa-speaking pastoralists engage in several different types of livestock transfers, each of which follows a different set of rules. In addition to bridewealth payments, osotua, and esile, these include *enkitaaroto*, in which animals are put in someone else’s herd but without a transfer of ownership, *ketaaro* or *elipa*, in which a milking cow is lent to a household in need, *aitogaroo*, in which a bull is lent for breeding purposes, and *keitapashaki*, in which animals are exchanged immediately, usually so that an individual can obtain a steer for ceremonial purposes (Perlov 1987:173–188).

Here we focus solely on osotua and esile, which have very different underlying logic and rules. When osotua partners transfer livestock, no debt is created, and it is inappropriate to talk about either debt or payment. Osotua partners have obligations to help one another, but the flow of goods and services between them does not need to be even roughly balanced over time (Cronk 2007). In transfers following the rules of esile, debt and repayment are of the essence. Esile means debt, and repayment is expected in the form of an animal at least as valuable if not more so than the one given. The repayment is referred to as elaata, which means to set free or untie a knot (Perlov 1987:184). If a debtor fails to repay, his creditor has the option of forgiving the debt but then referring to him henceforth as “Pasile”: One whose debt I have forgiven. This type of construction, in which the prefix “pa” is used to indicate what a person has given or received, is common in Maa, but it is normally used in a positive way. For example, a man refers to his father-in-law as “Pakiteng,” meaning “cow receiver.” The use of the term “Pasile” essentially serves as a mild public reproach to those who fail to repay their debts. If debts are not repaid before the debtor dies, they are passed on to his heirs.

In principle, risk pooling could be accomplished via a variety of different resource transfer rules. Here we focus on two rules that can lead to the pooling of risk: osotua and esile. Because many pastoralists other than the Maasai also have rules that are equivalent to osotua and esile (Almagor 1978; Bollig 1998, 2010; Dyson-Hudson 1966; Flannery *et al*. 1989; Gulliver 1955), in an effort to generalize our terminology we will henceforth refer to these not by their Maa labels but rather as “need-based transfers” and “account keeping.” Here we examine the underlying logic of both account keeping and need-based transfers and use an agent-based model to compare them in terms of their ability to enhance survival in volatile environmental conditions.

## Model

This model is adapted from Aktipis *et al*.’*s* (2011) agent-based model of risk pooling among Maasai pastoralists. That model was constructed to examine herd survival in volatile ecological conditions characteristic of East African pastoralism (Dahl and Hjort 1976; Homewood 2008). Here, in addition to incorporating account-keeping rules, we also generalize this model by exploring a wider variety of ecological conditions. All parameter values and assumptions about resource volatility were initially drawn from Aktipis *et al*. (2011) and Dahl and Hjort (1976), but were then varied to investigate our questions of interest.

**Fig. 1:**
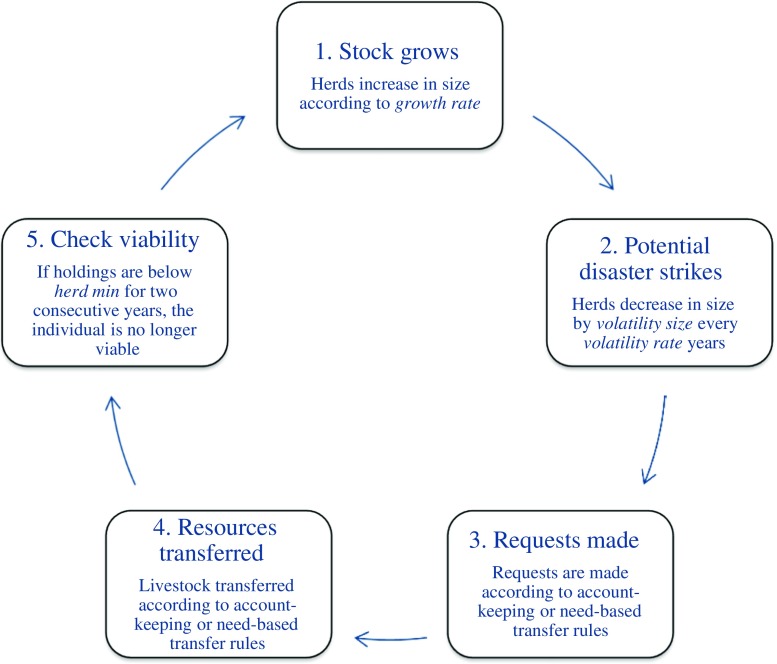
Overview of model schedule. The full model schedule is included in the appendix

We used Netlogo software to model a population of two actors, each with a herd of finite size. Each actor represented a household/family of approximately six individuals and began with a herd of 70. Although Maasai and other Maa-speaking pastoralists keep a variety of different types of livestock (cattle, goats, sheep, donkeys, and, in some arid areas, camels), in an effort to keep our model simple and tractable we refer simply to “stock.” Given that the Maasai economy is dominated by cattle, it would make the most sense for the reader to think of the “stock” in our model as cattle. During each time period, each actor’s resource stock grew or shrank at a rate normally distributed around a mean of 3.4 %, a typical annual growth rate (Dahl and Hjort 1976). Maximum herd size was 600, a realistic maximum herd size for an average sized household. During each period there was a chance that each herd would suffer a loss through drought or disease. As in Aktipis *et al*. (2011), we also ran additional simulations in which we varied the volatility size from zero to 50 % and the volatility rate from zero to 20 %. Based on estimates of a family’s caloric needs and productivity in the dry season (Dahl and Hjort 1976), we set the minimum size of a viable herd at 64. Although this is high compared to the living standards of some Maa-speaking pastoralists (e.g., Cronk 2004), the exact number is not important so long as it is consistent across all conditions. Importantly, this figure is also consistent with Aktipis *et al*. (2011) (Fig. 1 and Appendix).

We then simulated account-keeping-based and need-based livestock transfers between individuals. In order to establish a baseline for comparison, we first ran simulations in which no transfers occurred. For runs involving the transfer of livestock, we simulated interactions among individuals of the same type as well as individuals of different types.

Below we describe the algorithms underlying need-based transfers and account keeping. Account keeping requires the tracking of debt and credit over time while need-based transfers require individuals only to know their own resource holdings.

Need-based transfer rules were implemented as follows (consistent with Aktipis *et al*. 2011):

1. **Need**-**based asking rule**: Individuals ask their partners for livestock only if their current holdings are below the asking threshold (i.e., the minimum stock size of 64).
2. **Need**-**based giving rule**: Individuals give what is asked, but not so much as to put their herds below the giving threshold (also the minimum stock size of 64).

Account-keeping rules were implemented as follows:

1. **Account**-**keeping payback rule**:
  - If livestock have been previously transferred from the partner to the actor and the actor has enough to pay back without going below sustainability threshold (*resource min*), the actor ‘pays back’ livestock to his partner according to the actor’s *repayment probability*
2. **Account**-**keeping partner credit check rule**:
  - Checks whether partner is in good standing, which includes not having exceeded *tolerated delay* or *credit size* (when applicable)
3. **Account**-**keeping asking rule**:
  - As with the need-based transfer asking rule, individuals ask their partners for livestock if their current herd size is below the sustainability threshold of 64.
4. **Account**-**keeping giving rule**:
  - Response to partner- If a request is made, actors give if two conditions are met:
    i. If no debt remains from a previous request and partner is in good standing (meaning that previous debt had not existed for longer than *tolerated delay*)
    ii. The amount transferred cannot exceed the *credit size* extended to the partner

We then compared the performance of pairs of need-based transfer individuals with other types of pairs including no exchange pairs, account-keeping pairs and mixed pairs. Additional details regarding the model schedule, parameter values and model design can be found in the appendix.

## Results

### Survival of Need-Based-Transfer Pairs vs. Account-Keeping Pairs

**Fig. 2:**
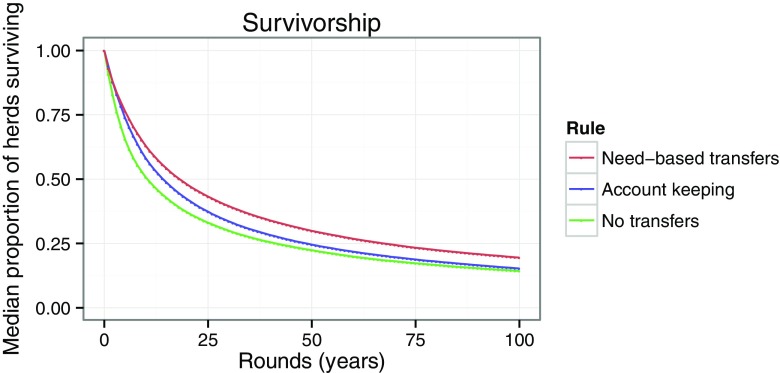
Need-based transfer pairs (*red*) show higher overall survival than account-keeping pairs (*blue*). Both need-based transfers and account keeping have higher survival than pairs in which no exchange occurs (*green*)

We compared median survival of pairs of need-based transfer individuals, account-keeping individuals and no-exchange individuals under the ecological conditions specified in the model description. We found that pairs of need-based transfer individuals had higher rates of herd survival than account-keeping pairs or pairs in which partners did not transfer resources (Fig. 2).

### Correlations of Survival Durations Within Pairs

**Fig. 3:**
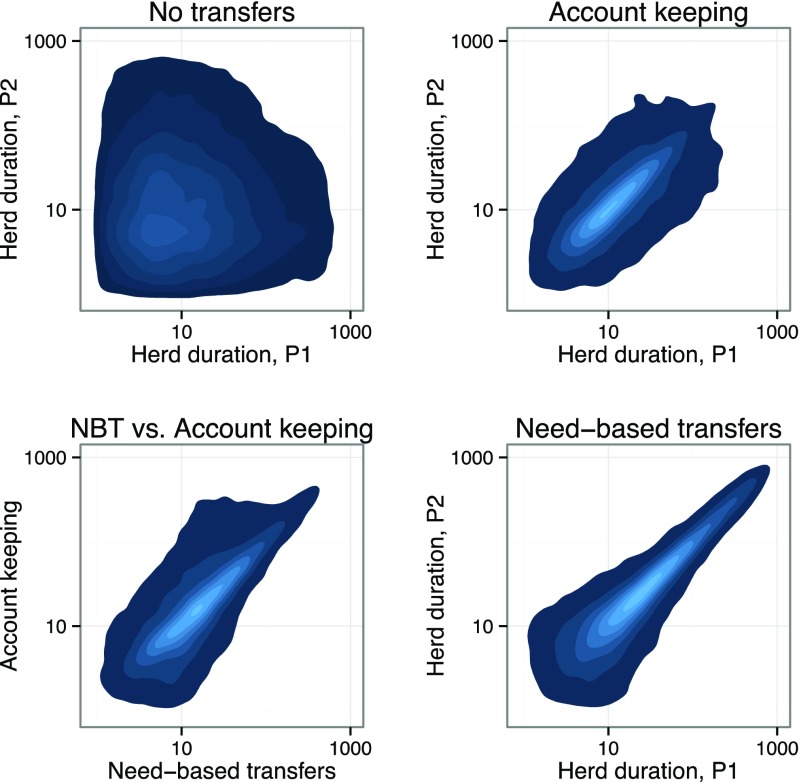
Pairs of need-based transfer individuals (*bottom right*) show greater correlations of herd survival durations than account-keeping pairs, no-exchange pairs or mixed pairs. Spearman’s rank correlations, *n* = 10,000 per condition: no-transfer condition ρ = −0.01, n.s.; account-keeping ρ = 0.40, p ≈ 0; heterogeneous strategies ρ = 0.45, p ≈ 0; need-based transfers ρ = 0.54, p ≈ 0. *p*-values for all transfer conditions < 10^−16^

In order to better understand whether individuals pool risk more effectively under need-based transfer rules than under account-keeping rules, we compared the median herd survival experienced by one individual in a simulation to that experienced by the other individual in the same simulation under four different conditions: (1) no exchange, (2) two account-keeping agents, (3) one account-keeping and one need-based transfer agent, and (4) two need-based transfer agents (Fig. 3). We found no correlation between survival durations when individuals did not make transfers, but highly significant correlations between the fates of the individuals when they did transfer livestock. Specifically, when both individuals used need-based transfer rules, correlations of their survival were higher (ρ = 0.54) than when both individuals used account-keeping rules (ρ = 0.40). This indicates that, in addition to being more effective than account-keeping at keeping livestock alive, need-based transfers also lead to a tighter yoking of the fates of the two parties in the risk-pooling relationship.

### Effects of Varying Environmental Volatility on Survival

**Fig. 4:**
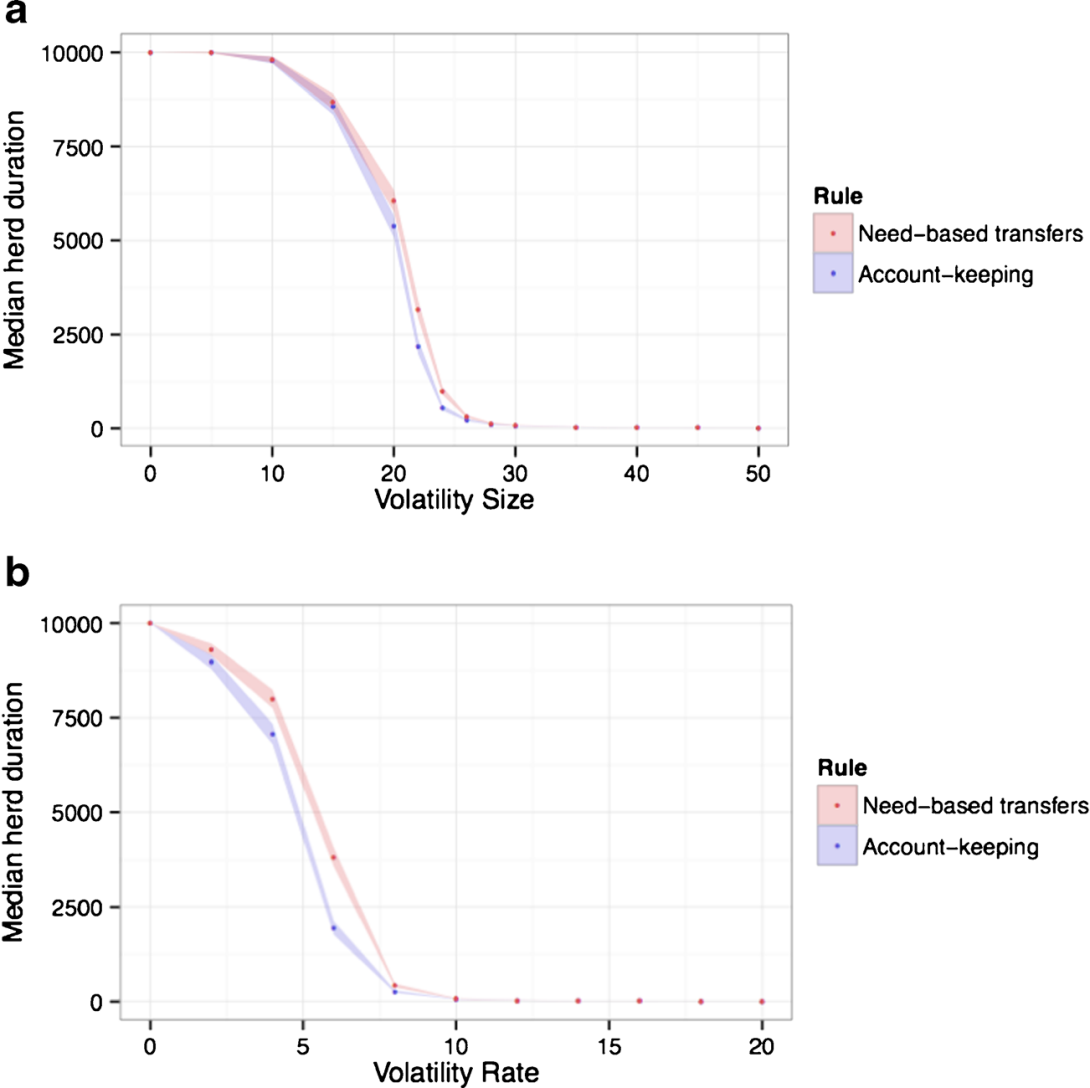
**a** Need-based transfers outperform account keeping most clearly when volatility size is between 20-30 % of total holdings. **b** Similarly, need-based transfers have the greatest advantage over account keeping when volatility rate is between 5-10 % per year. Shaded regions represent 95 % confidence intervals

In our baseline model, the likelihood and severity of losses were normally distributed around 30 % and 10 %, respectively. Given that the frequency of droughts in parts of East Africa has been increasing (Homann *et al*. 2008; Riché *et al*. 2009), we also looked at what happens when we vary the volatility size and volatility rate across simulations for pairs of account-keeping individuals and pairs of need-based transfer individuals (Fig. 4). When volatility is very low, all individuals survive and when it is very high all individuals die. At all values located between those two extremes, need-based transfer individuals survive longer than account-keeping individuals.

### Effects of Generosity on Survival

**Fig. 5:**
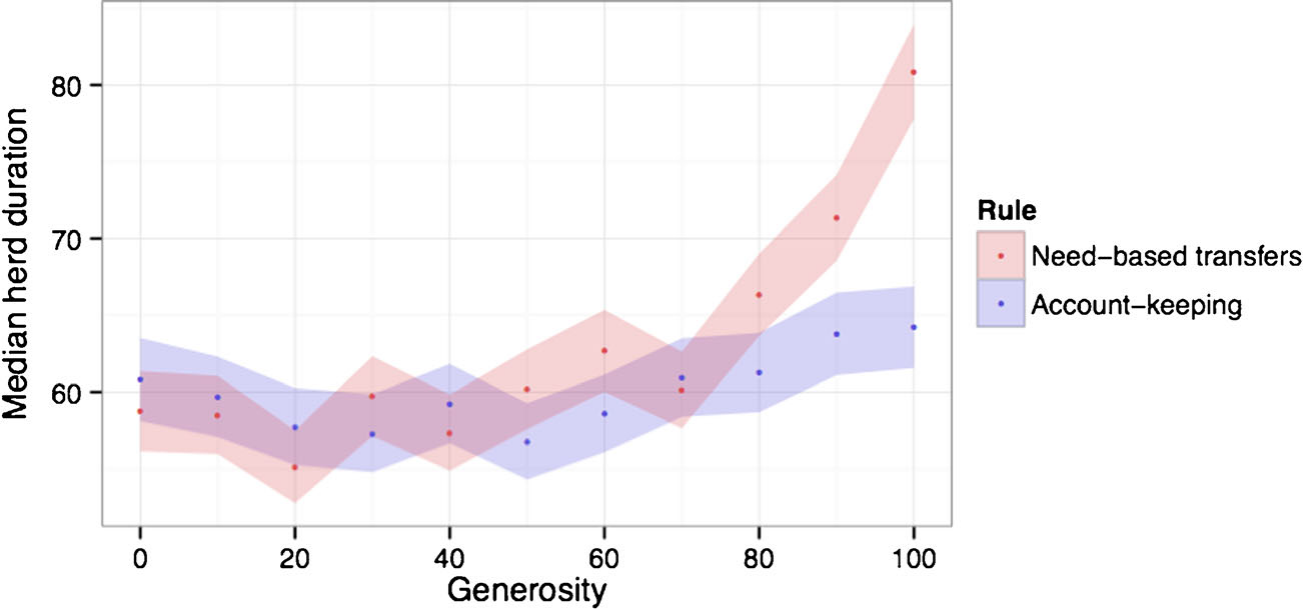
Need-based transfer pairs outperform account-keeping pairs only when generosity is 80 % or higher. Generosity is the likelihood of giving if asked by one’s partner and one is able (*need*-*based transfers*) or to a partner in good standing (*account keeping*). Shaded regions represent 95 % confidence intervals

In the osotua system, need-based transfers in their ideal form are always generous, i.e., individuals always give if they are asked and can do so without going below their own sustainability thresholds. We investigated whether the success of the need-based transfer rule was critically reliant on having 100 % generosity (Fig. 5). We varied the generosity of need-based transfer individuals and account-keeping individuals to compare the viability of these strategies. For need-based transfer individuals, generosity was the likelihood of giving if asked and able. For account-keeping individuals generosity was the likelihood of giving to a partner in good standing if asked. We found (1) that neither the account-keeping rule nor the need-based transfer rule consistently outperforms no exchange unless generosity is at least 80 % and (2) that, when generosity is high, pairs using the need-based transfer rule outperformed pairs using the account-keeping rule. Without generosity, need-based transfers are no more successful than account keeping.

### Survival in Mixed Pairs

**Fig. 6:**
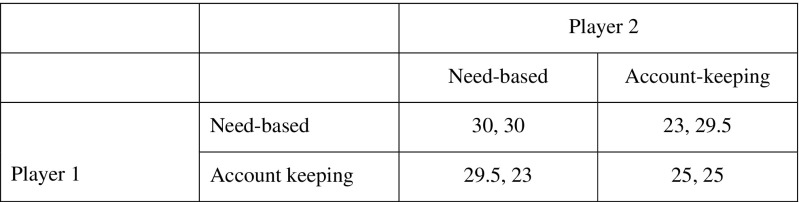
Payoffs in terms of survival at 50 years (in %) for individual and partner using each rule in ecologically realistic environmental conditions. The need-based transfer strategy is dominant to the account-keeping strategy

Under ecological conditions characterized by resource volatility, pairs using a need-based transfer strategy can outperform pairs using an account-keeping strategy. The relationship between the need-based transfer and account-keeping strategies is similar to the Stag Hunt Game (Skyrms 2003). Both strategies are coordination points, but two individuals using the need-based transfer strategy are likely to survive longer than two individuals using the account-keeping strategy (Fig. 6). The situation is thus a coordination problem that can be solved if all the individuals know both that there is a solution and that everyone involved also knows the solution (Chwe 2001; Cronk and Leech 2013). In the real world, common knowledge about need-based transfers can easily be provided by sharing norms such as the osotua concept that focus on the recipient’s need rather than on debt and repayment.

### Wealth Inequality

**Fig. 7:**
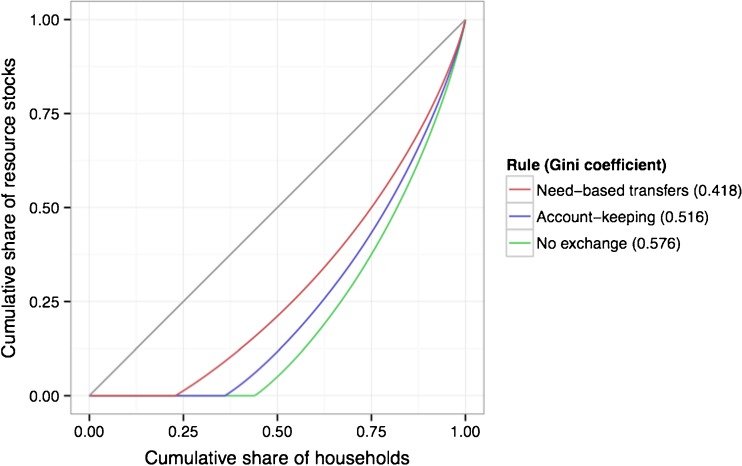
Stable differences in inequality exist between transfer strategies. Individuals engaging in no transfers (*green*) have the highest level of inequality, followed by account-keeping individuals (*blue*) and then dyads composed of need-based transfer individuals (*red*). The straight line (*gray*) shows perfect equality

In our model, stochastic growth and volatility of resources create wealth inequality. Inequality is often measured using the Gini coefficient (Gini 1912), which varies from 0 (when wealth holdings are equal) to 1 (when one person controls all the wealth). For comparative purposes, the highest Gini coefficient in the world is currently 0.632 (Lesotho), and lowest is 0.230 (Sweden). The figure for the United States is 0.45 (Central Intelligence Agency 2013). We find that need-based transfer individuals have the lowest inequality compared to all other individuals (Fig. 7). Wealth inequality is highest in the absence of wealth transfers (.576), followed by account-keeping individuals (.516), and need-based transfer individuals (.418). The Gini coefficient for need-based transfers (.418) is similar to that found for a sample of pastoralist societies (.42 ± .05) in a recent analysis of wealth inequality in societies with subsistence economies (Borgerhoff Mulder *et al*. 2009).

## Discussion

Inspired by the Maasai osotua system, Aktipis *et al*. (2011) used an agent-based model to test whether a need-based transfer rule leads to risk pooling and enhanced survival in volatile ecological conditions. Here we extended this model to test whether need-based transfer rules also outperform account-keeping in risky environments. We found that need-based transfers lead to greater risk pooling, longer survival and greater wealth equality than account-keeping, though both strategies outperformed scenarios involving no transfers of resources.

### Need-Based Transfers and Balanced Reciprocity

Reciprocity has been an important topic in economic anthropology since the days of Malinowski (1922), Mauss (1967), and Polanyi (1957). A common framework for understanding different types of reciprocity is Sahlins’ (1965) trichotomy of generalized, balanced, and negative reciprocity. Balanced reciprocity corresponds fairly closely with what we have been calling “account keeping.” We chose “account keeping” over balanced reciprocity in order to emphasize and capture the importance of debt and repayment in Maasai esile transfers. Our focus has been on the contrasts between account-keeping reciprocity and need-based transfers. Both of these patterns may be adaptive responses to individual needs that are asynchronous among individuals. When individuals’ needs all occur at the same time, neither account-keeping nor need-based transfers beyond close kin would likely be adaptive. But when needs arise asynchronously, it may make good adaptive sense for those with resources to transfer them to those without. The difference between need-based transfers and account-keeping becomes apparent when we consider the predictability of the needs in question. When needs are both asynchronous and highly predictable, account-keeping makes sense. If I know that I will always be in need on Tuesday and you know that you will always be in need on Friday, we can easily set up a system of balanced, tit-for-tat, account-keeping reciprocity that benefits us both. But if needs are not only asynchronous but also unpredictable, need-based transfers may, as our model suggests, make more adaptive sense than account-keeping. In short, when information about future needs is good, account-keeping may reign, while need-based transfers may prevail when such information is poor or nonexistent, as it was in our model. In developed economies, unpredictable needs are dealt with largely via formal insurance markets. Insurance companies overcome the problem of poor information about each individual’s needs by gathering large amounts of data about needs (rates of car accidents, home fires, etc.) of many people. That allows them to focus on the accuracy of data about needs in the aggregate rather than on the inaccuracy of data regarding each individual’s needs, which in turns allows them to set rates and engage in account-keeping exchanges with their clients (Levy 2012).

### Need-Based Transfers and Generalized Reciprocity

In Sahlins’ generalized reciprocity, sharing is indiscriminate and widespread. The same idea is also captured by Fiske’s “communal sharing” (Fiske 1991). Generalized reciprocity and communal sharing are good descriptions of how resources are often shared within households and within hunter-gatherer bands (e.g., Howell 2010; Woodburn 1998; Price 1975). However, neither is an accurate description of osotua relationships or other stock friendship relationships among pastoralists. Such relationships are characterized not by indiscriminate and widespread sharing but rather by limited contractual commitments between individual livestock owners. The advantage of the phrase “need-based transfers” is that it captures both of these patterns, which focuses our attention on the fact that risk-pooling results from both of them. This, in turn, provides a link between the study of need-based transfers and the existing literatures on risk-pooling in both anthropology (Bird and Bird 1997; Bliege Bird *et al*. 2002; Cashdan 1985; Gurven *et al*. 2000; Gurven and Hill 2009, 2010; Wiessner 1982; Winterhalder 1986) and economics (e.g., Barr and Genicot 2008; Fafchamps and Lund 2003).

### Computational Simplicity of Need-Based Transfers

In addition to its effectiveness in pooling risk, need-based transfer systems also have low cognitive load. Need-based transfer rules are simple: Ask if you need, give if you can. The rules underlying a tit-for-tat, account-keeping strategy can be straightforward in a Prisoner’s Dilemma framework (Axelrod 1984), but they prove much more complex in the more realistic situation we sought to model. In contrast to the simple rules followed by the need-based transfer agents, the account-keeping agents must follow a complex set of rules regarding such issues as credit, debt and repayment. Account-keeping agents use memory of their past transactions with other agents, whereas need-based transfer agents simply need to keep track of whether their own resource holdings are above their survival threshold and occasionally calculate whether they can afford to help a partner in need. The low cognitive requirements of need-based transfer systems suggest that they may have predated account-keeping in our species’ evolutionary history and could be more phylogenetically widespread than systems requiring account-keeping.

### Cheating and the Evolution of Cooperation

Organisms who live socially have the ability to manage risk through risk pooling, enabling them to live in more challenging ecological conditions by sharing resources during times of need. However, as with other explanations for the evolution of cooperation, the problem of cheating must be addressed.

The vast literature on cheater detection and cheater suppression has largely been motivated by solving the problem of cheating in the context of account-keeping interactions (Cosmides and Tooby 1992; Van Lier *et al*. 2013). Cheating in the context of need-based transfer is different, however, from cheating in an account-keeping system. In an account-keeping system, cheating is typically a matter of not repaying one’s debts. In a need-based transfer system, unbalanced accounts do not constitute cheating. Rather, cheating in a need-based transfer system involves asking for help when one is not in need or refusing to give when one is able. These different criteria for what constitutes cheating are another important way in which need-based transfers differ from account keeping.

Need-based transfers can be conceptualized as a form of decentralized and informal insurance. One of the problems that can arise when individuals have insurance in general is the problem of moral hazard, i.e., when individuals become more prone to take risks and act less carefully because they do not bear all the costs associated with a bad outcome. In this sense, the moral hazard problem may be at the heart of another way of cheating in a need-based transfer system: if being responsible and careful is costly, one can cheat by being lazy and taking unwise risks, knowing that one will receive help if the outcome is a severe loss or catastrophe. Interestingly, in the Maasai osotua system, individuals are also expected to act with responsibility, restraint and respect in how they handle their herds (Cronk 2007). In other words, they are expected to behave in such a way that may help to solve the moral hazard problem and minimize this type of cheating that is possible in systems of need-based transfers.

Another way of suppressing cheating is for individuals to carefully choose partners with whom they enter into need-based transfer relationships. Partner choice is one way of enhancing assortment of cooperators with one another, and it can be realized through both simple and complex rules for choosing and maintaining relationships (Aktipis 2004, 2006, 2011; Barclay 2013; Barclay and Willer 2007; Nesse 2009; Noe and Hammerstein 1994). Among the Maasai, need-based transfer relationships are taken very seriously and are said to be unbreakable once formed. Partner choice mechanisms could be at work in relationship formation if individuals can evaluate others through observing their behavior or reputations before entering into relationships in which they are committed to help. Choosing need-based transfer partners that have complementary risk profiles (i.e., independence of shocks) and responsible practices has high stakes. Discerning partner choice is therefore likely to play an important role in the viability of need-based transfer systems.

Need-based transfer systems require that individuals stay committed to helping a partner if that partner is unlucky; however, there may be conditions under which it is acceptable to terminate a need-based transfer relationship. These conditions may allow for dissolving relationships with greedy, stingy or irresponsible individuals (though not unfortunate ones). Relationship dissolution rules could thus provide another partner choice mechanism that could reduce cheating in need-based transfer systems. This is a research question that we will address in future fieldwork and modeling.

Finally, cheating in need-based transfer systems may be made difficult simply by the public nature of certain kinds of wealth. For example, among foragers, the same kinds of foods that are the most variable from day to day and the most likely to be widely shared (i.e., large game animals) are also the ones that are the most difficult to conceal. Among Maasai and other pastoralists, wealth primarily takes the form of livestock, whose visibility may make it difficult for anyone to feign either need or an inability to help. Despite the visibility of livestock, it would in principle be possible to hide one’s wealth by taking advantage of practices such as *enkitaaroto*, a Maasai system in which animals are put in someone else’s herd but without a transfer of ownership. We have livestock census data from two East African pastoralist societies, the Mukogodo Maasai of Kenya (Cronk 1989, 2004) and the Karimojong of Uganda. In both cases, the correlation between herders’ apparent wealth, defined as the numbers of animals in their herds regardless of who really owns them, and actual wealth, defined as the number of animals that they actually own regardless of whose herd they happen to be in, is too high for this kind of cheating to be a problem (Mukogodo Maasai: Pearson’s r = 0.984, *p* < 0.01, *N* = 183; Karimojong: Pearson’s r = 0.968, *p* < 0.01, *N* = 44). In systems where resources can be hidden or individuals are otherwise unable to evaluate the resource holdings of others, cheating in need-based transfer systems is likely to be a larger problem.

## Conclusion

Effectively managing risk and uncertainty are recurring adaptive problems across human societies. One way of managing the risks associated with life in volatile ecologies is to pool risk with others. Here we show that need-based transfers outperform account-keeping rules and can be effective even when implemented in computationally simple terms.

## Appendix

### Model Description

The model description offered below follows the standardized ODD protocol for describing individual and agent based models (Grimm *et al*. 2006) and is based on Aktipis *et al*. (2011).

#### Purpose

Here we use an agent-based model of wealth transfers within ecologically realistic conditions to investigate the viability of two sets of cooperative rules: one characterized by account keeping and the other characterized by risk pooling norms of need-based transfers. We then investigate how these two rules affect overall resource stock survivorship and the variability of survivorship within dyads.

#### State Variables and Scales

In this model time is represented discretely. Space is not explicitly modeled. Resource stock growth dynamics and volatility are implemented with global variables while the resource stock size and giving/asking rules are agent variables (Table 1). During each time period, agents execute the commands described in the schedule.

**Table 1:** Overview of state variables associated with each entity

Entity	State variable	Description
*Global*	• Growth rate	Amount by which resource stocks grow each year
	• Volatility rate	Likelihood of a negative event (e.g., drought)
	• Volatility size	Decrease in resource stock size resulting from negative event
	• Min. resource stock size	The minimum viable resource stock size
	• Max. resource stock size	The maximum resource stock that can be maintained
*Agents*	• Resource stock size • Net received	Number of resources in agent’s resource stock Number of resources received minus given to partner
	• Asking threshold	The threshold below which agents ask for resources
	• Generosity	The likelihood of giving if asked and able (need-based) or giving to partner in good standing (account-keeping)
*Need*-*based only*	• Giving threshold	The threshold below which agents will no longer give resources
*Account*-*keeping only*	• Credit size	The amount of credit granted to partner
	• Tolerated delay	The number of periods an agent will tolerate not being repaid by partner before placing partner in bad standing
	• P good standing	If partner is in good standing, means that they have not exceeded tolerated delay in past transfers
	• Repayment probability	The likelihood of repaying partner during each time period

#### Process Overview and Scheduling

This model proceeds in discrete time steps, and entities execute procedures according to the following ordering:

1. For each actor, resource stocks change in size:

- Resource stocks increase in size according to *growth rate*
- Resource stocks decrease in size by *volatility size* (as a percent of total holdings) according to *volatility rate*
- If *resource stock size* is above *resource stock max* it is set to *resource stock max*
- *Resource stock size* is rounded to nearest integer
  2. Requests are made:
  - If giving is need-based, requests are made if *resource stock size* is below *resource stock min*
  - If giving is account-keeping-based, requests are made if *resource stock size* is below *resource stock min*
    3. Transfers are made:
    - If giving is need-based, requests are fulfilled to the extent possible keeping the *resource stock size* of the giver above *resource stock min*
    - If giving is account-keeping-based
      - If resources have been previous transferred from the partner to the actor, the actor transfers *net received* resources to their partner according to *repayment prob*
      - If a new request was made, actors give if two conditions are met
        - The debt has not existed for longer than *tolerated delay*
        - The amount transferred cannot exceed the *credit size* extended to the partner.
    - All actors update *net received* to reflect transfers
      4. Actors removed from the population if two consecutive rounds occur where resources holdings are below *resource stock min*.
      5. *Age* of actors incremented by 1

#### Design Concepts

##### Emergence

In this model, risk pooling emerges from interactions between agents.

##### Prediction

Agents in this model lack the ability to predict outcomes of future environmental variability or future social interactions. They do not integrate information across time periods.

##### Sensing

Agents receive requests from their interaction partners and are able to examine their own resource holdings before fulfilling requests.

##### Interaction

Agents interact by making and fulfilling requests for resources.

##### Stochasticity

Resource stock growth and environmental volatility both have stochastic components.

##### Observation

Reported data are averaged from 10,000 runs. Simulations were run until both agents were removed from the population (i.e., dropped below the viability threshold for more than 2 consecutive time periods).

#### Initialization

All runs were initialized according to default parameters in the table below (Table 2).

**Table 2:** Initial and default values for all variables

Entity	State variable	Initial/Default value	Units
*Global*	• Growth rate	3.4 (SD: 2.53)	% current resource stock
	• Volatility rate	10	% per year
	• Volatility size	30 (SD: 10)	% of current resource stock
	• Min. resource stock size	64	Number resources
	• Max. resource stock size	600	Number resources
*Agents*	• Resource stock size	70	Number resources
	• Net received	0	Number resources
	• Asking threshold	64	Number resources
	• Generosity	100	% likelihood
*Need*-*based only*	• Giving threshold	64	Number resources
*Account*-*keeping only*	• Credit size	5	Number resources
	• Tolerated delay	5	Years
	• P good standing	Yes	Yes/No
	• Repayment probability	100	% likelihood

#### Input

In order to make our model of the Maasai pastoral system as realistic as possible, the following parameter values and assumptions about resource dynamics were based on existing scholarship (Dahl and Hjort 1976).

##### Growth rate

We used a 3.4 % growth rate with an SD of 2.53 based on Dahl and Hjort’s (1976:66) estimate the growth rate in “normal” conditions to be 3.4 %, with a maximum possible growth rate of roughly 11 % and a minimum of approximately −6 % (in the diminishing resource stocks example). Dahl and Hjort estimates are based on both empirical evidence and analytical modeling.

##### Resource stock size

Initial resource stock sizes in our model were 70, with a minimum of 64 and a maximum of 600. These values were derived from Dahl and Hjort (1976:178) who state that a resource stock of 64 resources is sufficient to sustain a reference family. Resource stock sizes described in the text range from 60–100 cows and resource stocks larger than 600 are not considered viable (Dahl and Hjort 1976:158).

##### Volatility

We used a volatility rate of .1, meaning that on average a disaster (e.g., drought or disease) occurred every 10 years. In our model, this disaster reduced the resources resource stock by 30 % on average, with a SD of 10 %. Dahl and Hjort (1976:114–130) note that these disasters occur approximately every 10–12 years based on empirical data, and that the population decline (during disasters that occur every 10 years) should not be more than approximately 28 %, based on analytical models.
